# Epidemiological data on systemic lupus erythematosus in native sub-Saharan Africans

**DOI:** 10.1016/j.dib.2019.104909

**Published:** 2019-11-27

**Authors:** Mickael Essouma, Jan René Nkeck, Francky Teddy Endomba, Jean Joel Bigna, Madeleine Singwe-Ngandeu, Eric Hachulla

**Affiliations:** a Department of Internal Medicine and Specialties, Faculty of Medicine and Biomedical Sciences, University of Yaoundé I, Yaoundé, Cameroon; b Division of Microbiology and Immunology, Doctoral School of Health Sciences, Faculty of Medicine and Biomedical Sciences, University of Yaoundé I, Yaoundé, Cameroon; c Psychiatry Internship Program, University of Bourgogne, 21000, Dijon, France; d Department of Epidemiology and Public Health, Centre Pasteur of Cameroon, Yaoundé, Cameroon; e School of Public Health, Faculty of Medicine, University of Paris Sud XI, Le Kremlin-Bicêtre, France; f Rheumatology Unit, Yaoundé Central Hospital, Yaoundé, Cameroon; g Department of Internal Medicine and Clinical Immunology, CHU Lille, University of Lille, 59037, Lille, France

**Keywords:** Systemic lupus erythematosus, Autoantibodies, Treatments, Outcomes, Native sub-Saharan Africans

## Abstract

Multiethnic studies conducted outside sub-Saharan Africa identify African Black people as the highest-risk group for morbidity and mortality among the 5,000,000 people who are affected by lupus globally. In the meantime, there have bee few attempts to summarize lupus data from sub-Saharan africa. We therefore conducted a systematic review and meta-analysis addressing systemic lupus erythematosus in Native sub-Saharan Africans. This paper both serves as repository for and describes the data obtained by qualitative and quantitative synthesis, notably the pooled prevalence of autoantibodies, the pooled frequency of cumulative drug use, the prevalence of comorbidities/complications and the mortality rate in Native sub-Saharan Africans with systemic lupus erythematosus. These data are interpreted in the research article titled “Systemic lupus erythematosus in Native sub-Saharan Africans: a systematic review and meta-analysis” (Essouma et al., 2019) [1].

**Table:** Specifications Table

Subject	Medicine and Dentistry
Specific subject area	Immunology, Allergology and Rheumatology
Type of data	Data presented in tables and figures
How data were acquired	Systematic literature search
Data format	Raw and analyzed data
Parameters for data collection	We collected data regarding both included studies (methods, setting, period, systemic lupus erythematosus prevalence, characteristics, drugs and outcome) and articles where these data were published (year of publication, name of the first author, journal)
Description of data collection	The above-mentioned data were extracted from the full-texts of eligible articles and cross-checked to ensure that there was no missing information
Data source location	Faculty of Medicine and Biomedical Sciences University of Yaoundé I Yaoundé Cameroon
Data accessibility	All data are included in this article
Related research article	Systemic lupus erythematosus in Native sub-Saharan Africans: a systematic review and meta-analysis. Mickael Essouma, Jan René Nkeck, Francky Teddy Endomba, Jean Joel Bigna, Madeleine Singwe-Ngandeu, Eric Hachulla, J Autoimmun 2019 (In press) [1]

**Table:** 

**Value of the Data** • This article permits an in-depth understanding of data on systemic lupus erythematosus in Native sub-Saharan Africans. • These data are beneficial for health professionals and researchers involved in systemic lupus erythematosus management and research, as well as local health authorities. • As these data inform on the burden and management of systemic lupus erythematosus in Native sub-Saharan Africans, they may be used to increase awareness for systemic lupus erythematosus in sub-Saharan Africa and serve as accurate basis for building capacity for research and management of systemic lupus erythematosus in Native sub-Saharan Africans.

## 1 Data description

**Fig. 1:**
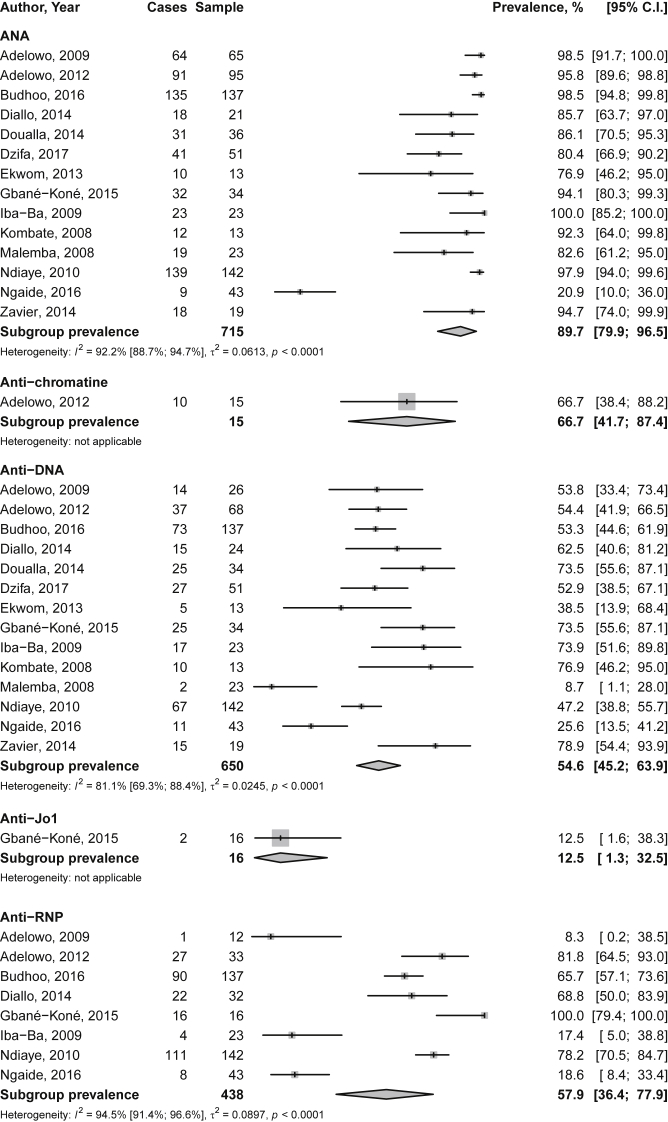

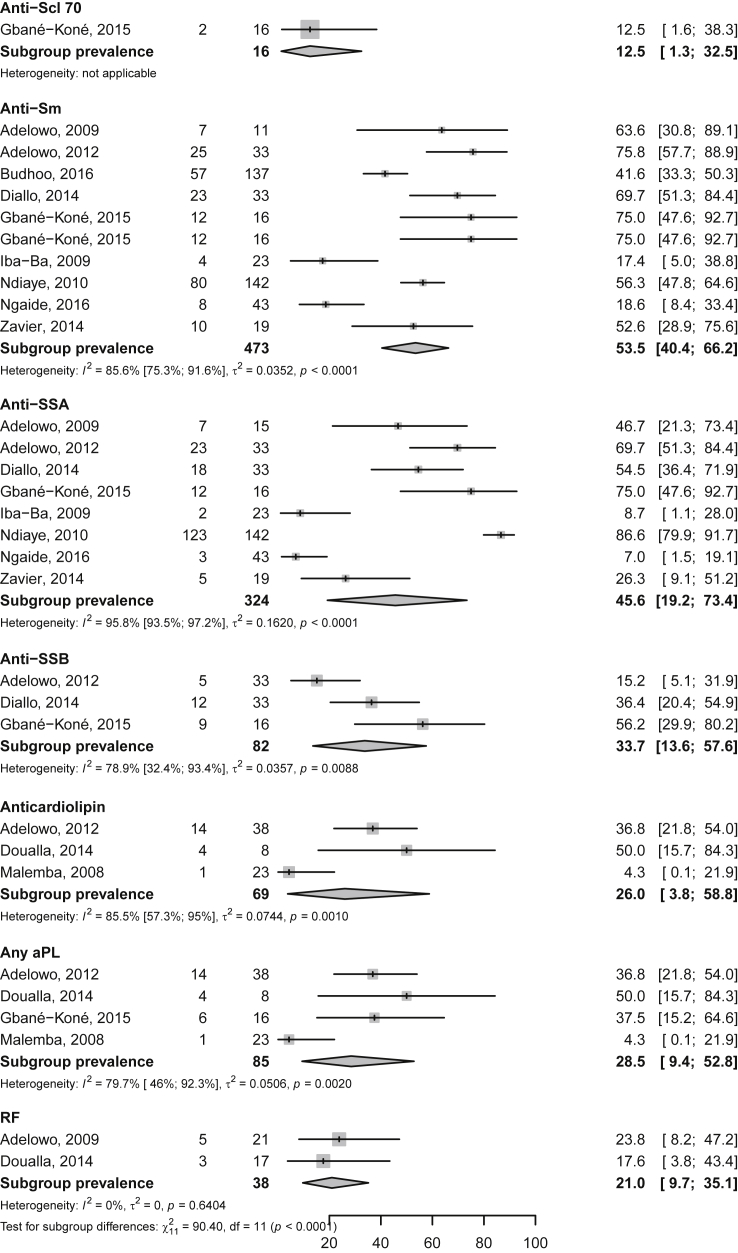
Prevalence of autoantibodies in Native sub-Saharan Africans with systemic lupus erythematosus. Grey boxes represent the effect estimates (prevalence), and the horizontal bars represent the 95% confidence intervals (CI). The size of the boxes is proportional to the inverse variance. The diamonds are for the pooled effect estimates and 95% CI, and the dotted vertical line has been added to assist visual interpretation. ANA antinuclear antibodies; anti-DNA anti-deoxyribonucleic acid; anti-RNP anti-ribonucleoprotein; anti-Sm anti-Smith; anti-SSA anti-Sjogren syndrome antigen A; anti-SSB anti-Sjogren syndrome antigen B; aPL antiphospholipid antibodies; RF rheumatoid factor.

**Fig. 2:**
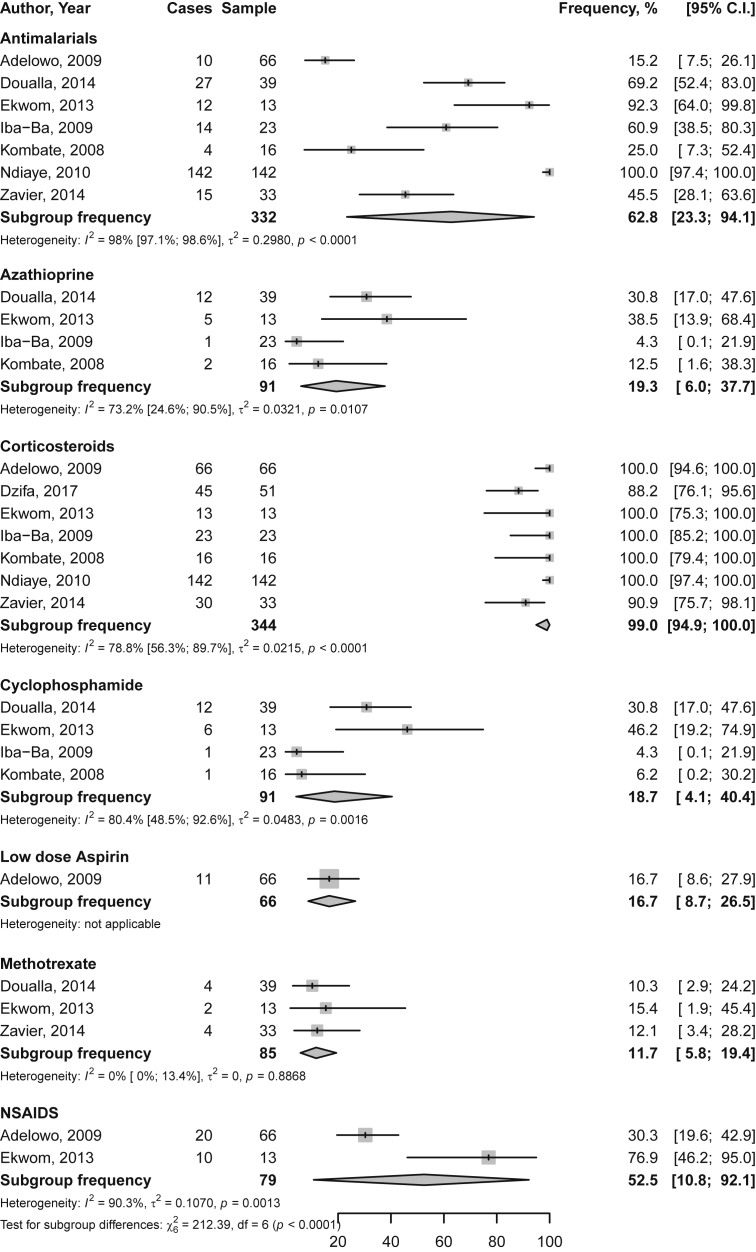
Frequency of cumulative drug use among Native sub-Saharan Africans with systemic lupus erythematosus. Grey boxes represent the effect estimates (frequency), and the horizontal bars represent the 95% confidence intervals (CI). The size of the boxes is proportional to the inverse variance. The diamonds are for the pooled effect estimates and 95% CI, and the dotted vertical line has been added to assist visual interpretation.

**Fig. 3:**
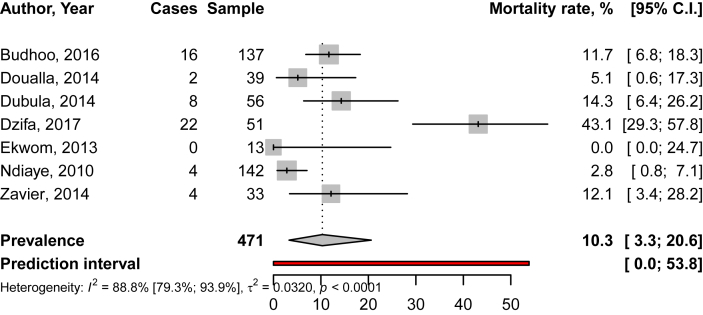
Mortality rate in Native sub-Saharan Africans with systemic lupus erythematosus. Grey boxes represent the effect estimates (prevalence), and the horizontal bars represent the 95% confidence intervals (CI). The size of the boxes is proportional to the inverse variance. The diamonds are for the pooled effect estimates and 95% CI, and the dotted vertical line has been added to assist visual interpretation.

**Fig. 4:**
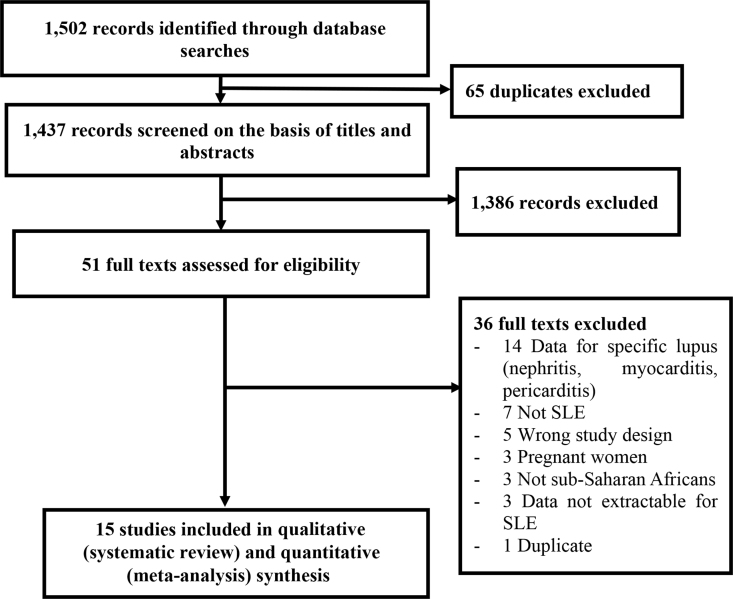
PRISMA flow chart of study selection. SLE systemic lupus erythematosus.

We herein report the pooled prevalence rates of autoantibodies (Fig. 1), the pooled frequencies of cumulative drug use (Fig. 2), the prevalence of comorbidities/complications (Table 1) and the pooled mortality rate (Fig. 3). The main search strategy used (in PUBMED) to obtain these data is displayed in Table 2 and Fig. 4 describes the study selection process. Table 3 summarizes the characteristics of the overall 15 included studies [[2], [3], [4], [5], [6], [7], [8], [9], [10], [11], [12], [13], [14], [15], [16]] whereas Table 4 summarizes only the studies included in the mortality analysis [4,[6], [7], [8], [9],^14^,^16^].

**Table 1:** Prevalence of comorbidities and complications in Native sub-Saharan Africans with systemic lupus erythematosus.

Complications/comorbidities	Prevalence, range %
Infections [5,6,8,9,11,12]	4.3–68.7
Cardiovascular diseases and risk factors	
-Heart failure [8]	33.3
-Stroke [6,10,12]	5.1–6.8
-Peripheral vein thrombosis [8,11]	2–4.3
-Diabetes mellitus [6,12]	5.1–18.7
-Hypertension [2,6,9]	10.3–19.6
Chronic kidney disease [6,10,12,16]	6.2–9.4
Any aseptic osteonecrosis [6,10]	2.6–6.2

**Table 2:** Main search strategy for PubMed.

Search	Search terms
#1	“systemic lupus erythematosus” OR “disseminated lupus erythematosus” OR SLE OR DLE OR “lupus nephritis” OR “renal SLE” OR “cutaneous lupus” OR “cutaneous DLE” OR “Lupus Erythematosus Disseminatus” OR “Libman-Sacks Disease” OR “Lupus vasculitis”
#2	Africa* OR Algeria OR Angola OR Benin OR Botswana OR “Burkina Faso” OR Burundi OR Cameroon OR “Canary Islands” OR “Cape Verde” OR “Central African Republic” OR Chad OR Comoros OR Congo OR “Democratic Republic of Congo” OR Djibouti OR Egypt OR “Equatorial Guinea” OR Eritrea OR Ethiopia OR Gabon OR Gambia OR Ghana OR Guinea OR “Guinea Bissau” OR “Ivory Coast” OR “Cote Ivoire” OR Jamahiriya OR Kenya OR Lesotho OR Liberia OR Libya OR Madagascar OR Malawi OR Mali OR Mauritania OR Mauritius OR Mayotte OR Morocco OR Mozambique OR Namibia OR Niger OR Nigeria OR Principe OR Reunion OR Rwanda OR “Sao Tome” OR Senegal OR Seychelles OR “Sierra Leone” OR Somalia OR “South Africa” OR “St Helena” OR Sudan OR Swaziland OR Tanzania OR Togo OR Tunisia OR Uganda OR “Western Sahara” OR Zaire OR Zambia OR Zimbabwe OR “Central Africa” OR “Central African” OR “West Africa” OR “West African” OR “Western Africa” OR “Western African” OR “East Africa” OR “East African” OR “Eastern Africa” OR “Eastern African” OR “North Africa” OR “North African” OR “Northern Africa” OR “Northern African” OR “South African” OR “Southern Africa” OR “Southern African” OR “sub Saharan Africa” OR “sub Saharan African” OR “subSaharan Africa” OR “subSaharan African”
#3	#1 AND #2

**Table 3:** Summary of studies addressing systemic lupus erythematosus in Native sub-Saharan Africans in 2008–2018.

Study	Design	Country	Setting	Locality	Period of recruitment	Number of participants	Number of participants with SLE	Classification criteria for SLE	Females, n (%)	Mean age at diagnosis of SLE, y	Age range, y	Study quality
Adelowo. 2009 [9]	Cross-sectional	Nigeria	Hospital based	Urban	2001–2006	1250	66	1982 ACR	63 (95.5)	33	16–60	Moderate
Adelowo. 2012 [10]	Cross-sectional	Nigeria	Hospital based	Urban	2001–2010	95	95	1982 ACR	91 (95.7)	33.4	17–55	Low
Budhoo. 2016 [11]	Cross-sectional	South Africa	Hospital based	Urban	2003–2012	137	137	1997 ACR	125 (91.2)	32.2	NR	Low
Diallo. 2014 [12]	Cross-sectional	Senegal	Hospital based	Urban	2010–2012	35	35	1997 ACR	33 (94.3)	32.8	18–50	Low
Doualla. 2014 [13]	Cross-sectional	Cameroon	Hospital based	Urban	1999–2009	6485	39	1997 ACR	36 (92.3)	39.2	19–59	Moderate
Dubulla. 2014 [14]	Cross-sectional	South Africa	Hospital based	Urban	2003–2009	56	56	1982 ACR and 1997 ACR	51 (91.2)	30.3	NR	Low
Dzifa. 2017 [15]	Cohort	Ghana	Hospital based	Urban	2007–2009	51	51	1982 ACR	45 (86.5)	30.4	14–68	Moderate
Ekwom. 2013 [16]	Cross-sectional	Kenya	Hospital based	Urban	2010–2011	394	13	1982 ACR and 1997 ACR	13 (100)	34	12–52	High
Gbané-Koné. 2015 [17]	Cross-sectional	Ivory Coast	Hospital based	Urban	1987–2014	18,076	117	1982 ACR	115 (98.3)	35.8	12–73	Moderate
Iba-Ba. 2009 [18]	Cross-sectional	Gabon	Hospital based	Urban	2004–2008	23	23	1982 ACR and 1997 ACR	22 (95.6)	32.8	18–68	Moderate
Kombate. 2008 [19]	Cross-sectional	Togo	Hospital based	Urban	1991–2003	16	16	1997 ACR	16 (100)	31.9	15–46	Low
Malemba. 2008 [20]	Cross-sectional	Congo, RD	Hospital based	Urban	1988–2002	2370	23	1982 ACR	21 (91.3)	31.8	NR	Low
Ndiaye. 2008 [21]	Cross-sectional	Senegal	Hospital based	Urban	1997–2006	142	142	1982 ACR and 1997 ACR	125 (88)	34	6–72	Low
Ngaidé. 2016 [22]	Cross-sectional	Senegal	Hospital based	Urban	2011–2012	50	50	1997 ACR	46 (92)	36.2	14–60	Moderate
Zavier. 2014 [23]	Cross-sectional	Benin	Hospital based	Urban	2000–2013	33	33	1997 ACR	32 (97)	28.8	16–51	Low

SLE systemic lupus erythematosus; ACR American College of Rheumatology; n number; y years; NR not reported; Congo RD Democratic Republic of the Congo.

**Table 4:** Summary of studies reporting a mortality rate in Native sub-Saharan Africans with systemic lupus erythematosus.

Study	Design	Country	Duration of SLE	Duration of follow up	Mortality rate	Study quality
Dzifa. 2017 [8]	Cohort	Ghana	Mean 25.2 ± 31.5 months 1–143	Mean 26.1 ± 26.6 days (1–140)	43.1	Moderate
Dubula. 2014 [7]	Cross-sectional	South Africa	Median 8 months (IQR, 1–61)	3–106 days	14.3	Low
Budhoo. 2016 [4]	Cross-sectional	South Africa	Median 42 months (IQR, 22–88.3)	Median 36 months (IQR, 12.5–68)	11.7	Low
Zavier. 2014 [16]	Cross-sectional	Benin	NR	NR	12.1	Low
Doualla. 2014 [6]	Cross-sectional	Cameroon	NR	NR	5.1	Moderate
Ndiaye. 2010 [14]	Cross-sectional	Senegal	NR	10 days-117 months	2.8	Low
Ekwom. 2013 [9]	Cross-sectional	Kenya	1–12 months	1–12 months	0.0	High

SLE systemic lupus erythematosus; IQR interquartile range; NR not reported.

## 2 Experimental design, materials, and methods

• Searched databases and search strategy

A comprehensive search of PubMed, Excerpta Medica database (EMBASE), Web of Science, African Journals Online, and Global Index Medicus was conducted to identify all relevant articles published from January 1, 2008 to October 7, 2018, without any language restriction. We considered recent studies to have the current and updated clinical overview of systemic lupus erythematosus in the region. We conceived and applied a search strategy based on the combination of relevant terms. The main search strategy in PubMed was adapted for the search in the other databases. A manual search that consists of scanning reference lists of eligible studies and relevant reviews was performed to identify any studies missed during the review process or by the search strategy.

The titles and abstracts of the retrieved papers were independently screened by two investigators (ME and JRN) and the full-texts of papers deemed potentially eligible were further assessed for final inclusion. All discrepancies for study selection were resolved through discussion or with the arbitrage of a third investigator.

• Criteria for considering studies for the review
➢ Types of studies

Observational studies including cross-sectional, case-control and cohort studies, as well as case series. We did not consider case reports, commentaries, review articles and letters to the editor.

➢ Types of participants

We considered studies involving African Black people (or multiethnic groups with possibility to extract information for the African Black people) living in sub-Saharan Africa regardless of the age and gender. Studies were excluded if: (1) they included multiethnic groups with no possibility to extract informations regarding only the African Black people (2) they only included a specific group of lupus patients i.e. lupus nephritis, neuropsychiatric lupus, cutaneous lupus, lupus pericarditis, lupus myocarditis, lupus in pregnant women (3) they included patients with overlapping syndromes.

➢ Condition

The classification for systemic lupus erythematosus was based on the 1982 American College of Rheumatology and/or revised 1997 American College of Rheumatology criteria [17,18].

➢ Outcomes of interest

The following outcomes were analyzed: systemic lupus erythematosus prevalence; demographic, clinical and immunological characteristics of systemic lupus erythematosus; frequencies of cumulative drug use for the treatment of systemic lupus erythematosus and its complications; outcome measures of systemic lupus erythematosus.

• Data extraction and management

The data were extracted by two investigators (ME and JJB) using a preconceived, piloted and standardized data abstraction form. The following data were extracted and cross-checked to ensure that there was no missing information: name of the first author, year of publication, study design, period of recruitment of the study population, setting (country, unique/multiple site[s]), locality (urban/rural), sampling method, systemic lupus erythematosus diagnostic criteria and the outcomes of interest.

• Assessment of the methodological quality of studies

We used an adapted version of the tool developed by Hoy and colleagues [19] to assess the methodological quality of included studies. Three investigators (JJB, ME and FTAE) independently ran the assessment. Discrepancies were discussed and resolved by these investigators. Cohen's κ statistics were used for inter-rater agreements between investigators regarding study inclusion and for the assessment of the methodological quality of the included studies.

• Data synthesis and analysis

The quantitative synthesis was done using the ‘*meta*’ packages of the R statistical software (version 3.5.1, The R Foundation for statistical computing, Vienna, Austria). We used the reference method for prevalence synthesis suggested by Barendregt and colleagues [20]. The prevalence of systemic lupus erythematosus and systemic lupus erythematosus autoantibodies, the frequencies of cumulative drug use and the mortality rate were recalculated based on crude numerators and denominators provided by individual studies. To minimize the effect of studies with extremely small or extremely large prevalence estimates on the overall estimate, the variance of study-specific prevalence was stabilized with the Freeman-Tukey double arcsine transformation before pooling the data with the random effects meta-analysis model [20]. Heterogeneity was assessed by the chi-square test on Cochrane's Q statistic, and quantified by I^2^ values. Low, moderate and high heterogeneity were considered for I^2^ values of 25%, 50% and 75% respectively. The quality of the included studies is described in Table 3. The Egger's test was used to assess the presence of publication bias, and a statistically significant publication bias was considered for *p*-values < 0.1. We decided a priori that if we find publication bias, we will do no adjustment in regard, since we believed that the prevalence estimates of interest would likely be published even if they are substantially different from the previously reported estimates.
